# Prenatal Triclosan Exposure and Anthropometric Measures Including Anogenital Distance in Danish Infants

**DOI:** 10.1289/ehp.1409637

**Published:** 2016-02-23

**Authors:** Tina Harmer Lassen, Hanne Frederiksen, Henriette Boye Kyhl, Shanna H. Swan, Katharina M. Main, Anna-Maria Andersson, Dorte Vesterholm Lind, Steffen Husby, Christine Wohlfahrt-Veje, Niels E. Skakkebæk, Tina Kold Jensen

**Affiliations:** 1 Department of Growth and Reproduction, Rigshospitalet, Copenhagen University Hospital, Copenhagen, Denmark; 2 Hans Christian Andersen Children’s Hospital, Odense University Hospital, Odense, Denmark; 3 Odense Patient data Exploratory Network (OPEN), Odense University Hospital, Odense, Denmark; 4 Department of Preventive Medicine, Icahn School of Medicine at Mount Sinai, New York, New York, USA; 5 Department of Environmental Medicine, Institute of Public Health, University of Southern Denmark, Odense, Denmark

## Abstract

**Background:**

Triclosan (TCS) is widely used as an antibacterial agent in consumer products such as hand soap and toothpaste, and human exposure is widespread. TCS is suspected of having endocrine-disrupting properties, but few human studies have examined the developmental effects of prenatal TCS exposure.

**Objectives:**

We prospectively examined associations between prenatal TCS exposure and anthropometric measures at birth and anogenital distance (AGD) at 3 months of age.

**Methods:**

Pregnant women from the Odense Child Cohort (n = 514) provided urine samples at approximately gestational week 28 (median 28.7 weeks, range 26.4–34.0), and urinary TCS concentration was measured by isotope dilution TurboFlow–liquid chromatography–tandem mass spectrometry. Multiple linear regression analysis was used to examine associations between prenatal TCS exposure and measures of size at birth (birth weight, length, head and abdominal circumference) and AGD at 3 months of age (median 3.3 months, range 2.3–6.7 months), controlling for potential confounders.

**Results:**

Newborn boys in the highest quartile of prenatal TCS exposure had a 0.7-cm [95% confidence interval (CI): –1.2, –0.1, p = 0.01] smaller head circumference than boys in the lowest quartile. Additionally in boys, inverse associations of borderline statistical significance were observed between prenatal TCS exposure and abdominal circumference at birth and AGD at 3 months of age (p-values < 0.10). Prenatal TCS exposure was not significantly associated with any of the outcomes in girls. However, AGD was measured in fewer girls, and we observed no significant interactions between a child’s sex and prenatal TCS exposure in anthropometric measures at birth.

**Conclusion:**

Prenatal TCS exposure was associated with reduced head and abdominal circumference at birth and with reduced AGD at 3 months of age in boys, although the last two findings were statistically nonsignificant. These findings require replication but are compatible with an anti-androgenic effect of prenatal TCS exposure on fetal growth in boys.

**Citation:**

Lassen TH, Frederiksen H, Kyhl HB, Swan SH, Main KM, Andersson AM, Lind DV, Husby S, Wohlfahrt-Veje C, Skakkebæk NE, Jensen TK. 2016. Prenatal triclosan exposure and anthropometric measures including anogenital distance in Danish infants. Environ Health Perspect 124:1261–1268; http://dx.doi.org/10.1289/ehp.1409637

## Introduction

Triclosan (TCS) is a biocide used as an antibacterial and antifungal agent in a number of consumer products such as toothpaste, mouthwash, disinfectants, and soaps (Dann and Hontela 2011). Evidence from *in vitro* and animal studies suggests endocrine-disrupting properties of TCS including antiandrogenic activity and disturbance of thyroid hormone action (Ahn et al. 2008; Axelstad et al. 2013; Gee et al. 2008; Kumar et al. 2009; Paul et al. 2010, 2012; Veldhoen et al. 2006). Human exposure to TCS is widespread, and studies in pregnant women have found detectable levels of TCS in the vast majority of the participants (Casas et al. 2011; Philippat et al. 2014; Wolff et al. 2008). Moreover, TCS has been detected in amniotic fluid, indicating that TCS can enter the fetal environment through placental transfer (Philippat et al. 2013). Fetal life is considered a particularly vulnerable period for exposure to endocrine-disrupting chemicals because hormonal disturbances during organ development may introduce irreversible changes (Drake et al. 2009; MacLeod et al. 2010; van den Driesche et al. 2011; Welsh et al. 2008). However, little is known about the potential adverse effects of human environmental exposure to TCS during fetal life (Wolff et al. 2008; Philippat et al. 2012, 2014). A statistically nonsignificant inverse association between boys prenatally exposed to TCS and birth length was reported in a cohort study from the United States (Wolff et al. 2008). In a French study of 520 male newborns, prenatal triclosan exposure was inversely associated with prenatal growth parameters measured by ultrasound at approximately gestational week 33 and was statistically nonsignificantly associated with reduced head circumference at birth (Philippat et al. 2014).

Anogenital distance (AGD), the distance from the anus to the genitals, is sexually dimorphic, with males having a 50–100% longer AGD than females (Hsieh et al. 2008; Salazar-Martinez et al. 2004; Swan et al. 2015). In rodents, the AGD has been shown to be determined by fetal androgen action during early stages of fetal development (Welsh et al. 2008). Thus, a reduced AGD in males may be indicative of insufficient testosterone during the early stages of development of male reproductive organs, whereas an increased AGD in females suggests excessive androgen exposure during the early stages of development of female reproductive organs (Welsh et al. 2008). In humans, a reduced AGD has been observed among boys with the genital malformations hypospadias and cryptorchidism (Hsieh et al. 2008, 2012; Jain and Singal 2013). Moreover, prenatal exposure to bisphenol A and phthalates has been associated with reduced AGD in human male infants (Bornehag et al. 2015; Bustamante-Montes et al. 2013; Miao et al. 2011; Suzuki et al. 2012; Swan 2008; Swan et al. 2005, 2015). Little is known about the relationship between the length of the AGD in females and female reproductive system characteristics (Mendiola et al. 2012). We have not been able to identify any human studies examining associations between prenatal exposure to TCS and AGD.

Because very little is known about the potential effects of prenatal TCS exposure in humans, the aim of our study was to examine the associations between maternal urinary excretion of TCS as a measure of prenatal TCS exposure and birth outcomes as well as measurement of AGD at 3 months of age stratified by child sex.

## Materials and Methods

### Study Population

The study was based on data from the Odense Child Cohort (Kyhl et al. 2015). Briefly, newly pregnant women residing in the municipality of Odense, Denmark between 1 January 2010 and 31 December 2012 were recruited at gestational age 8–16 weeks at a voluntary informational meeting about ultrasound examinations, at the first antenatal midwife visit, or at the ultrasound examination at Odense University Hospital. In total, 6,707 pregnant women were eligible for the study, although only 4,017 were informed about it. As of November 2014, 2,874 women (42.9% of the total number eligible) were enrolled in the cohort (Kyhl et al. 2015).

While recruitment to the Odense Cohort was ongoing, TCS measurements were obtained from urine samples collected at approximately week 28 of pregnancy (median 28.7 weeks, range 26.4–34.0) from a subset of women with singleton pregnancies (*n* = 565). The subset of women was selected based on the availability of urine samples. The first 196 samples were selected randomly from among the women enrolled in the Odense Child Cohort between September 2010 and June 2011, whereas the last 369 samples were selected from the remaining women who were enrolled by January 2012 who had available information from questionnaires, urine samples, birth records, and clinical examination of the child at 3 months of age. Of the 565 women, 51 women were excluded because of non-Caucasian origin (*n* = 30), missing information on ethnicity (*n* = 16), or missing data on child sex (*n* = 5), leaving 514 mother-child pairs (273 males and 241 females) eligible for analyses.

The study was approved by the local ethics committee, and the women gave written consent to participate in the study. The research was conducted in accordance with the principles of the Declaration of Helsinki.

### Birth Outcomes

From birth records, we obtained information about maternal prepregnancy BMI, gestational age (days) at birth, and birth measures such as birth weight (grams), length (centimeters), head circumference (centimeters) and abdominal circumference (centimeters).

### AGD and Penile Measurements

Three months after the expected date of birth, regardless of the actual gestational age at birth, the children were invited to a clinical examination (median age 3.3 months, range 2.3–6.7 months), which included measurements of length, weight, and AGD. In addition, genital malformations were noted. Two different measures of AGD were made using a Vernier caliper (SPI DigiMax) in both boys and girls: In girls, a short AGD was measured from the center of the anus to the posterior fourchette (AGDaf), and a long AGD was measured from the center of the anus to the top of the clitoris (AGDac). Correspondingly, in boys, a short AGD was measured from the center of the anus to the posterior base of the scrotum (AGDas), and a long AGD was measured from the center of the anus to the cephalad insertion of the penis (AGDap). Penile width was also measured using a Vernier caliper. In each child, the genital measures were repeated three times, and an arithmetic mean was calculated.

Among the 514 mother–child pairs, 252 males and 179 females had a minimum of one AGD measurement, and 250 males had a minimum of one penile measurement at the clinical examination at approximately 3 months of age. AGD measurements were initiated in August 2011, but owing to technical difficulties in measuring AGD in girls, these data were valid from October 2011. Therefore, data on AGD were available for fewer girls than boys. Because children with only one or two repeated genital measures or who had missing data on the included covariates were excluded, *n* = 245 (AGDas), *n* = 236 (AGDap), *n* = 241 (penile width), *n* = 178 (AGDaf), and *n* = 176 (AGDac) were included in the statistical analyses.

Four technicians measured AGD in both the boys and the girls. The coefficient of variation (CV) was < 10% for all the triplicate AGD measurements, except for AGDaf, in which two girls had CVs of 0.10 and 0.14. We conducted subanalyses from which those two girls were excluded. Additionally, we conducted subanalyses in which we also included children who had only one or two AGD measurements [*n* = 1 (AGDac), *n* = 1 (AGDas), *n* = 6 (AGDap), *n* = 3 (penile width)]. For those with two AGD measurements, the average was used as the outcome measure.

### TCS Measurements

At approximately week 28 of gestation, urine samples were collected in the morning from fasting pregnant women and were subsequently stored at ≤ –20°C in freezers at the Odense Patient data Explorative Network (OPEN) until chemical analyses of total (free and conjugated) TCS by isotope dilution TurboFlow–liquid chromatography–tandem mass spectrometry (LC-MS/MS) with preceding enzymatic deconjugation (Frederiksen et al. 2013a). Briefly, the 565 samples were analyzed in 17 batches. The first 196 samples were analyzed between December 2011 and January 2012, and the following 369 samples were analyzed approximately 1 year later at the end of 2012. TCS levels were similar in the 2011 and 2012 measurements (*p* = 0.44, assessed by one-way analysis of variance). Each batch included standards for calibration curves, approximately 35 unknown samples, 2 blanks, 2 urine pool controls, and 2 urine pool controls spiked with TCS standards at low and high levels. The interday variation, expressed as the relative standard deviation (RSD), was ≤ 14% for both spike levels. The recovery of spiked samples was > 77%. We used the same control materials during both measuring periods, and there was no difference in TCS concentration in the spiked urine control material. The level of detection (LOD) for TCS was 0.06 ng/mL. More details on urinary TCS excretion levels in the present cohort as well as levels of other phenols measured have recently been published by Frederiksen et al. (2014).

Urinary osmolality, which is a measure of urinary dilution, was measured by the freezing point depression method using an automatic cryoscopic osmometer (Osmomat® 030; Gonotec GmbH). For each ninth sample measurement, a urine pool was measured as a control. The mean urinary osmolality for this control pool (*n* = 77) was 0.825 osmoles/kilogram (Osm/kg) with a relative standard deviation (RSD) of 1.85%. The median (5th, 95th percentile) osmolality of all urine samples included in this study was 0.64 (0.209, 0.930) Osm/kg.

### Statistics

TCS concentrations were adjusted for urinary osmolality normalized to the median osmolality of all samples (0.64 Osm/kg) to correct for dilution of the urine. This adjustment was performed for all samples with a measured TCS concentration above the LOD by dividing the individual urinary TCS concentration (nanograms/milliliter) with the individual osmolality of the urine sample (osmoles/kilogram) and multiplying with the median osmolality of all samples (osmoles/kilogram) (Lassen et al. 2013). Urinary TCS concentrations below the LOD were not adjusted for osmolality but were substituted by LOD divided by the square root of 2. Osmolality-adjusted TCS [nanograms/milliliter_(osm)_] and the samples below the LOD were divided into sex-specific quartiles based on the distributions among the 273 males and among the 241 females. The osmolality-adjusted TCS concentrations and the samples below the LOD were also entered in the statistical model as a continuous variable and were log2-transformed because of skewed distribution. The birth outcomes (weight, length of the child, head circumference, and abdominal circumference), the AGD measurements, and the penile width were left untransformed because of acceptable normal distributions of the residuals.

We calculated the distribution of anthropometric measures at birth and the anogenital distance as well as the correlations (Spearman correlation coefficients) between the genital measures among the boys and among the girls. Differences in the distributions of the TCS concentrations according to population characteristics were assessed by one-way analysis of variance. Multiple linear regression analysis was used to analyze the associations between urinary TCS excretion and birth outcomes and AGD measurements adjusted for potential confounders. We tested for linear trends across TCS quartiles in regression models by means of ordinal TCS quartiles using integer values from 1 to 4. Confounders included in multivariable models were factors known *a priori* to be important predictors of birth outcomes or AGD. AGD measurements vary with the age and weight of the child, and because the clinical examination was scheduled to take place 3 months after the expected date of birth, we constructed a measure of “post-conceptional age,” which we defined as the sum of the gestational age at birth (days) and the age of the child at the AGD measurements (days). Analyses of associations between TCS and AGD were thus adjusted for the post-conceptional age and the individual weight-for-age standard deviation score (SD-score) (Swan 2008) calculated using Danish longitudinal growth data (Tinggaard et al. 2014). Additionally, to examine whether systematic differences in AGD measurements between the examiners could confound the association between TCS and AGD as outcome, we performed sensitivity analyses with inclusion of information on the examiner as a categorical variable with five categories (four different examiners and a category for missing information of examiner).

Analyses of associations between TCS and infant size at birth (birth weight, birth length, head circumference, and abdominal circumference) were adjusted for parity (primiparous/multiparous), maternal smoking during pregnancy (yes/no), prepregnancy BMI (< 20, 20–25, > 25 kg/m^2^) and gestational age (days). All results are presented stratified by sex given *a priori* interest in sex differences. However, we also tested for potential interaction between child sex and TCS exposure in models with birth weight, birth length, head circumference, and abdominal circumference as outcomes by inserting a product interaction term of child sex × maternal urinary TCS levels (continuous, osmolality-adjusted, log2-transformed TCS concentration) in the statistical models. Percentage change in outcomes was calculated as the difference between the highest exposed group and the reference group divided by the intercept × 100, where the reference values for the covariates were gestational age at birth of 280 days, prepregnancy BMI between 20 and 25 kg/m^2^, primiparous, and nonsmokers.

There are known ethnic differences in size at birth and AGD (Papadopoulou et al. 2013; Sathyanarayana et al. 2010); therefore, we excluded non-Caucasian women to avoid confounding because of ethnicity. However, we performed sensitivity analyses in which we included the non-Caucasian women. We also performed sensitivity analyses in which maternal height and prepregnancy weight were included in the statistical models instead of prepregnancy BMI.

Residual plots were used to examine the model assumption of homogeneity of variances, whereas the normality of the distribution of residuals was examined graphically using histograms and normal probability plots (data not shown). Associations were considered statistically significant at the *p* < 0.05 level, and observations with missing data for variables included in the statistical analyses were excluded. We focused on associations where we observed trends across quartiles because we considered single significant observations without a dose–response trend to be less reliable.

All data analysis was performed using SAS v.9.1 (SAS Institute Inc.).

## Results

The mean age at birth among the 514 women was 31.0 years, 55% of the women were primiparous, and 3.6% smoked during pregnancy. The mean gestational age at birth was 280 days (range 205–296 days) for girls and 280 days for boys (range 221–297 days). Detectable maternal urinary TCS concentration was found in 83% of the samples (girls: 84%, boys: 82%). The median unadjusted urinary TCS concentration was 0.88 ng/mL, and the 95th percentile and the maximum unadjusted TCS concentration were 428 ng/mL and 2,614 ng/mL, respectively. The median (5th, 95th percentile) osmolality-adjusted TCS concentrations stratified by child sex are shown in Table 1. No significant associations between maternal osmolality-adjusted TCS levels and population characteristics were observed in either girls or boys, and maternal osmolality-adjusted TCS levels were not significantly different between girls and boys. Data on covariates were missing for < 2.5% of the observations. The 514 women with TCS measurements eligible for this study did not differ significantly from the other singleton birth–giving women enrolled in the Odense cohort with respect to gestational age at delivery, maternal parity, and age (data not shown), whereas the mean birth weight among the children included in this study was statistically significantly higher (3,554 g vs. 3,495 g). There were fewer smokers in the present cohort, although this difference was not statistically significant (3.6% vs. 5.1%).

**Table 1:** Population characteristics according to median (5th, 95th percentile) osmolality-adjusted maternal urinary triclosan excretion [nanograms/milliliter_(osm)_] stratified by child sex among 514 mother-child pairs.

Population characteristics	Girls		Boys	
	*n*^*a*^ (%)	Median TCS (5th, 95th percentile) ng/mL_(osm)_	*n*^*b*^ (%)	Median TCS (5th, 95th percentile) ng/mL_(osm)_
All	241 (100)	1.01 (< LOD, 536)	273 (100)	0.96 (< LOD, 335)
Maternal age at birth
< 29 years	77 (32)	0.94 (< LOD, 536)	102 (38)	0.76 (< LOD, 210)
29–33 years	82 (34)	0.91 (< LOD, 474)	81 (30)	0.89 (< LOD, 160)
> 33 years	81 (34)	1.43 (< LOD, 562)	84 (31)	1.14 (< LOD, 669)
Prepregnancy BMI (kg/m^2^)
< 20	22 (9)	1.14 (< LOD, 20)	26 (10)	1.31 (< LOD, 538)
20–25	130 (54)	0.95 (< LOD, 503)	141 (53)	0.93 (< LOD, 182)
25+	88 (37)	1.07 (< LOD, 719)	100 (37)	0.91 (< LOD, 318)
Parity
Primiparous	124 (51)	1.06 (< LOD, 664)	156 (57)	0.98 (< LOD, 227)
Multiparous	117 (49)	0.95 (< LOD, 503)	116 (43)	0.88 (< LOD, 538)
Maternal smoking during pregnancy
Yes	9 (4)	0.94 (< LOD, 503)	9 (3)	0.31 (< LOD, 1.70)
No	231 (96)	1.06 (< LOD, 546)	258 (97)	0.97 (< LOD, 339)
Preterm birth^*c*^
Yes	8 (3)	1.04 (0.09, 664)	9 (3)	0.82 (< LOD, 23)
No	232 (97)	1.03 (< LOD, 536)	258 (97)	0.95 (< LOD, 339)
Abbreviations: BMI, body mass index; LOD, level of detection; TCS, triclosan. ^***a***^Because of missing data, the numbers do not add up to 241 for the covariates. ^***b***^Because of missing data, the numbers do not add up to 273 for the covariates. ^***c***^Birth before gestational week 37.				

Mean AGD measures and birth outcomes stratified by child sex are shown in Table 2. The correlations between the two different AGD measures in boys and in girls were *r* = 0.63 (*p* < 0.0001), and *r* = 0.61 (*p* < 0.0001), respectively. As expected, the mean AGD measures in the boys were nearly twice the corresponding AGD measures in the girls (Table 2). Penile width was weakly correlated with AGDas (*r* = 0.22, *p* = 0.0006) and AGDap (*r* = 0.14, *p* = 0.03).

**Table 2:** Distribution (mean, ± standard deviation) of anthropometric measures at birth and ­anogenital distance at approximately 3 months of age among boys and girls from the Odense Child Cohort.

Anthropometric and AGD measures	Girls			Boys		
	*n*	Mean	SD	*n*	Mean	SD
Birth weight (g)	240	3,502	503	267	3,600	536
Birth length (cm)	239	51.8	2.2	264	52.4	2.4
Head circumference (cm)	238	34.9	1.7	264	35.5	1.8
Abdominal circumference (cm)	237	33.5	2.3	261	33.7	2.1
AGD, short (mm)	179	20.1	3.6	251	36.8	5.5
AGD, long (mm)	177	37.7	4.4	242	70.7	6.5
Penile width (mm)	—	—	—	247	13.8	1.2
Abbreviations: AGD, anogenital distance; SD, standard deviation.						

### Associations Between Maternal Urinary TCS Levels and Size at Birth

**Figure 1:**
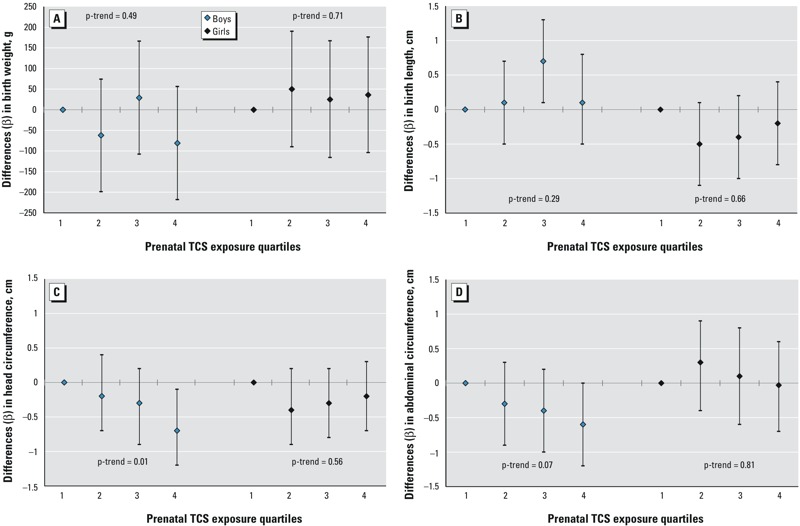
Differences (β-coefficients from multiple linear regression) and 95% confidence intervals (CIs) in anthropometric measures at birth among newborn boys and girls from the Odense Child Cohort in relation to prenatal triclosan (TCS) exposure quartiles [nanograms/milliliter_(osm)_]. All estimates are adjusted for gestational age, maternal smoking, parity, and prepregnancy body mass index (BMI). Median (range) for osmolality-adjusted TCS quartiles (ng/mL_osm_) in boys: 1st = < level of detection (LOD) (< LOD–< 0.24), 2nd = 0.53 (0.24–< 0.97), 3rd = 1.69 (0.97–< 3.24), 4th = 17.8 (3.24–1,702); in girls: 1st = < LOD (< LOD–< 0.247), 2nd = 0.50 (0.247–< 1.05), 3rd = 2.03 (1.05–< 4.21), 4th = 103.1 (4.21–2,350). *p*-Trend: *p*-value for trend across TCS quartiles.

Among the boys, we observed a linear dose-dependent inverse association between maternal urinary TCS levels and head circumference indicated by the trend across TCS quartiles (*p*-trend = 0.01); for every doubling in TCS concentration, head circumference decreased by 0.06 cm among the boys [95% confidence interval (CI): –0.11, –0.002, *p* = 0.04] (Table 3 and Figure 1). When maternal urinary TCS levels were categorized in quartiles, boys in the highest TCS quartile had, on average, a 0.7-cm (95% CI: –1.2, –0.1, *p* = 0.01) smaller head circumference than boys in the first quartile. This difference corresponds to a reduction of 2.0% (95% CI: –3.5%, –0.4%) between the fourth versus the first exposure quartile. Maternal urinary TCS level was also inversely associated with abdominal circumference among the boys in a dose-dependent manner, although not statistically significantly (*p*-trend = 0.07) (Table 3 and Figure 1). Boys in the highest TCS quartile had, on average, a 0.6-cm smaller abdominal circumference than boys in the first TCS quartile, although the estimate did not reach statistical significance (95% CI: –1.2, 0.0, *p* = 0.07). This difference corresponds to a reduction in mean abdominal circumference of 1.8% (95% CI: –3.6%, 0.1%). We observed no significant associations between prenatal TCS exposure and birth weight [e.g., for boys in the highest vs. lowest exposure quartile, β = –81 g (95% CI: –218, 56, *p* = 0.25)], and there was no consistent trend in estimated associations with increasing exposure. We observed a significant positive association between birth length and the third versus the first quartile of TCS, but in the other quartiles, the β values were close to null and without a consistent pattern according to prenatal TCS exposure (Table 3 and Figure 1).

**Table 3:** Associations between maternal pregnancy triclosan levels and birth outcomes among newborn boys and girls from the Odense Child Cohort.

TCS	Birth weight (g)			Birth length (cm)			Head circumference (cm)			Abdominal circumference (cm)		
	*n*	β (95% CI)	*p*-Value	*n*	β (95% CI)	*p*-Value	*n*	β (95% CI)	*p*-Value	*n*	β (95% CI)	*p*-Value
Boys
TCS quartile^*a*^
1st	69	Reference		69	Reference		69	Reference		68	Reference
2nd	66	–62 (–199, 74)	0.37	66	0.1 (–0.5, 0.7)	0.83	66	–0.2 (–0.7, 0.4)	0.52	64	–0.3 (–0.9, 0.3)	0.33
3rd	66	29 (–108, 166)	0.68	65	0.7 (0.1, 1.3)	0.03	64	–0.3 (–0.9, 0.2)	0.24	64	–0.4 (–1.0, 0.2)	0.22
4th	66	–81 (–218, 56)	0.25	64	0.1 (–0.5, 0.8)	0.65	65	–0.7 (–1.2, –0.1)	0.01	65	–0.6 (–1.2, 0.0)	0.07
*p*-Trend^*b*^			0.49			0.29			0.01			0.07
Continuous^*c*^	267	–5.2 (–18.5, 8.2)	0.45	264	0.01 (–0.05, 0.07)	0.70	264	–0.06 (–0.11, –0.002)	0.04	261	–0.05 (–0.11, 0.01)	0.09
Girls
TCS quartile^*a*^
1st	60	Reference		59	Reference		59	Reference		59
2nd	60	50 (–90, 190)	0.48	60	–0.5 (–1.1, 0.1)	0.11	60	–0.4 (–0.9, 0.2)	0.19	60	0.3 (–0.4, 0.9)	0.47
3rd	60	25 (–116, 167)	0.72	60	–0.4 (–1.0, 0.2)	0.24	60	–0.3 (–0.8, 0.2)	0.28	59	0.1 (–0.6, 0.8)	0.84
4th	60	36 (–104, 176)	0.62	60	–0.2 (–0.8, 0.4)	0.53	59	–0.2 (–0.7, 0.3)	0.49	59	–0.03 (–0.7, 0.6)	0.93
*p*-Trend^*b*^			0.71			0.66			0.56			0.81
Continuous^*c*^	240	4.7 (–7.3, 17.0)	0.43	239	–0.004 (–0.06, 0.05)	0.89	238	–0.01 (–0.05, 0.04)	0.70	237	0.00 (–0.06, 0.06)	0.998
TCS × Sex
*p*-Interaction^*d*^	507		0.39	503		0.57	502		0.24	498		0.27
Abbreviations: CI, confidence interval; LOD, level of detection; TCS, triclosan. All estimates are adjusted for gestational age, maternal smoking, parity and prepregnancy BMI. ^***a***^Median (range) for osmolality-adjusted TCS quartiles (nanograms/milliliter_(osm)_) in boys: 1st = < LOD (< LOD to < 0.24), 2nd = 0.53 (0.24 to < 0.97), 3rd = 1.69 (0.97 to < 3.24), 4th = 17.8 (3.24 to 1,702); in girls: 1st = < LOD (< LOD to < 0.247), 2nd = 0.50 (0.247 to < 1.05), 3rd = 2.03 (1.05 to < 4.21), 4th = 103.1 (4.21 to 2,350). ^***b***^*p*-Value for trend across TCS quartiles. ^***c***^log2-transformed TCS concentration. ^***d***^*p*-Value for interaction [child sex × maternal urinary TCS levels (continuous log2-transformed TCS concentration)]. Median gestational week of maternal urine collection for boys: 28.7 (26.4–30.4) weeks; for girls: 28.7 (range 27.9–34.0) weeks.												

We found no significant associations or consistent dose–response relationships between prenatal TCS exposure and any of the measures of size at birth in girls (Table 3 and Figure 1).

The interaction terms between child sex and prenatal TCS exposure in models with birth weight, birth length, head circumference, and abdominal circumference as outcomes were not statistically significant (Table 3), but the population size (i.e., the statistical power) was likely too small to test for interaction.

### Associations Between Maternal Urinary TCS Levels and AGD at 3 Months of Age

In the boys, inverse associations between maternal urinary TCS levels and both the long and the short AGD measures were observed, although the estimates did not reach statistical significance (*p*-values < 0.10): every doubling in TCS concentration was associated with a decrease of 0.2 mm (95% CI: –0.3, 0.0, *p* = 0.08) in AGDas and a decrease of 0.2 mm (95% CI: –0.4, 0.0, *p* = 0.05) in AGDap (Table 4). Penile width was not associated with maternal urinary TCS levels.

**Table 4:** Associations between maternal pregnancy TCS levels and AGDas (short AGD measure), AGDap (long AGD measure) and penile width in boys and AGDaf (short AGD measure) and AGDac (long AGD measure) in girls at 3 months of age from the Odense Child Cohort.

TCS quartile^*a*^	Boys									Girls					
	AGDas (mm)			AGDap (mm)			Penile width (mm)			AGDaf (mm)			AGDac (mm)		
	*n*	β (95% CI)	*p*-Value	*n*	β (95% CI)	*p*-Value	*n*	β (95% CI)	*p*-Value	*n*	β (95% CI)	*p*-Value	*n*	β (95% CI)	*p*-Value
1st	64	Reference		63	Reference		63	Reference		51	Reference		50	Reference	
2nd	61	–0.9 (–2.8, 0.9)	0.33	60	0.3 (–1.7, 2.3)	0.77	59	0.0 (–0.4, 0.5)	0.81	40	–0.5 (–2.0, 1.0)	0.51	40	–0.6 (–2.4, 1.2)	0.54
3rd	62	–2.3 (–4.1, –0.4)	0.02	59	–1.2 (–3.3, 0.8)	0.24	62	–0.1 (–0.5, 0.3)	0.55	43	–0.4 (–1.8, 1.1)	0.59	43	0.1 (–1.6, 1.9)	0.87
4th	58	–1.3 (–3.1, 0.6)	0.19	54	–1.3 (–3.4, 0.8)	0.22	57	–0.2 (–0.6, 0.3)	0.44	44	–0.3 (–1.8, 1.1)	0.66	43	0.1 (–1.7, 1.8)	0.95
*p*-Trend^*b*^			0.08			0.11			0.32			0.69			0.79
Continuous^*c*^	245	–0.16 (–0.34, 0.02)	0.07	236	–0.20 (–0.39, 0.00)	0.05	241	–0.02 (–0.06, 0.02)	0.27	178	–0.02 (–0.15, 0.10)	0.72	176	0.01 (–0.15, 0.17)	0.89
Abbreviations: AGD, anogenital distance; AGDac, long AGD measure (girls); AGDaf, short AGD measure (girls); AGDap, long AGD measure (boys); AGDas, short AGD measure (boys); CI, confidence interval; TCS, triclosan. All estimates are adjusted for weight for age standard deviation score and post-conceptional age (gestational age at birth + age of the child at the AGD measurements, days). ^***a***^Median (range) for osmolality-adjusted TCS quartiles (nanograms/milliliter_(osm)_) in boys: 1st = < LOD (< LOD to < 0.24), 2nd = 0.53 (0.24 to < 0.97), 3rd = 1.69 (0.97 to < 3.24), 4th = 17.8 (3.24 to 1,702); in girls: 1st = < LOD (< LOD to < 0.247), 2nd = 0.50 (0.247 to < 1.05), 3rd = 2.03 (1.05 to < 4.21), 4th = 103.1 (4.21 to 2,350). ^***b***^*p*-Value for trend across TCS quartiles. ^***c***^Log2-transformed TCS concentration. Median age at AGD measurements for boys, 3.3 months (range 2.3–6.2 months); for girls, 3.3 months (range 2.3–6.7 months).															

We observed no significant associations or consistent dose–response associations between prenatal TCS exposure and anogenital distance in the girls (although our calculations were based on fewer observations than in the boys) (Table 4).

Subanalyses showed that inclusion of children with only one or two of the three AGD measurements did not appreciably change the results (< 10% change in estimates; results not shown); nor did inclusion of examiner information, inclusion of non-Caucasian women, or inclusion of maternal height and prepregnancy weight instead of prepregnancy BMI in the analyses substantially change the observed associations (results not shown).

## Discussion

In this prospective study, we observed an inverse association between prenatal TCS exposure as measured by maternal urinary TCS excretion and head circumference in newborn boys. There were no clear associations between prenatal TCS exposure and birth weight or birth length in boys. We also observed reduced abdominal circumference and shorter AGD in boys born to mothers with higher urinary TCS levels, although these estimates did not reach statistical significance (*p* < 0.10). In contrast, we observed no significant associations between prenatal TCS exposure and any of the outcomes in girls. However, AGD was measured in fewer girls. Analyses of interactions between sex and prenatal TCS exposure were not statistically significant, which was likely because of small sample size and therefore low statistical power to perform interaction analyses.

The observed reduction of 0.7 cm in head circumference in boys between the highest versus the lowest exposed quartile was of similar magnitude to the differences in head circumference between boys and girls that were observed in this study (mean difference = 0.6 cm) as well as having been reported at birth in other studies (Greil 2006; Sankilampi et al. 2013). For example, in a Finnish register-based study based on 533,666 singletons born between 1996 and 2008, girls born at 40 weeks of gestation had on average a 0.65-cm smaller head circumference than boys born at the same gestational age (Sankilampi et al. 2013).

Few human studies have examined prenatal TCS exposure and birth outcomes, and to our knowledge, no human study has examined associations with AGD. Consistent with the findings of the present study, a French mother–child cohort study including 520 newborn boys observed a statistically nonsignificant inverse association between prenatal triclosan exposure and head circumference, but no associations with length or weight at birth were observed (Philippat et al. 2014). In a cohort study from the United States that included 339 mothers and their offspring, Wolff et al. (2008) reported a statistically nonsignificant inverse association between prenatal TCS exposure and birth length among boys. Consistent with the findings of the present study, Wolff et al. did not observe any associations between prenatal TCS exposure and birth outcomes among girls (Wolff et al. 2008). Notably, both of these earlier studies reported considerably higher median maternal urinary TCS concentrations [Wolff et al. (2008): 11 ng/mL; Philippat et al. (2014): 30 ng/mL] than those reported in our study (0.88 ng/mL). However, the variation in TCS levels in the present study was large (5th, 95th percentile and maximum: < LOD, 428, and 2,614 ng/mL, respectively), suggesting that some pregnant Danish women were as highly exposed as women in previous studies [Wolff et al. (2008): maximum TCS concentration, 1,790 ng/mL; Philippat et al. (2014): 95th percentile TCS concentration, 755 ng/mL]. The differences in overall urinary TCS excretion across studies may reflect differences in consumer behavior between countries.

Studies in rats have shown no effects of prenatal TCS exposure on size at birth or on AGD (Axelstad et al. 2013; Rodríguez and Sanchez 2010). However, reproductive effects, including reduced levels of follicle-stimulating hormone (FSH), luteinizing hormone (LH), and testosterone, have been shown in adult male rats exposed to 20 mg/kg bw/day TCS for a period of 60 days (Kumar et al. 2009), and reduced sperm production was observed in rats exposed to 50 and 200 mg/kg TCS for 8 weeks (Lan et al. 2013). These findings suggest that the hypothalamic–pituitary–gonadal axis may be affected by TCS exposure. Furthermore, in a number of rat studies, TCS exposure has consistently been shown to induce hypothyroxinemia in dams exposed during gestation and lactation (Axelstad et al. 2013; Paul et al. 2010, 2012) and in neonate pups exposed prenatally (Paul et al. 2010, 2012). These findings suggest that TCS may have thyroid hormone–disrupting effects. Additionally, *in vitro* studies have suggested different modes of action for TCS, including estrogenic, androgenic, anti-androgenic and gestagenic effects (Ahn et al. 2008; Christen et al. 2010; Gee et al. 2008; Schiffer et al. 2014).

This study was not designed to elucidate mechanisms of action. However, because we observed associations between prenatal TCS exposure and measures of growth only among the boys (although the results of interaction analyses were nonsignificant), and because the difference in mean head circumference between boys with the highest versus the lowest TCS exposure was comparable to the average difference in head circumference between boys and girls reported for other populations (Sankilampi et al. 2013), we speculate that our findings may be compatible with an anti-androgenic mechanism of action of TCS. Nevertheless, other mechanisms of action of TCS leading to diminished growth are also possible, such as a perturbation of the thyroid axis. These results need to be confirmed in future studies, ideally with measurement of hormone levels. A causal relationship between prenatal TCS exposure and reduced head circumference would be of considerable public health significance because reduced head circumference at birth has been associated with impaired cognitive performance (Lundgren et al. 2001; Veena et al. 2010).

A reduced AGD in males may be a marker of testicular dysgenesis syndrome (TDS) (Juul et al. 2014; Thankamony et al. 2014), suggesting that some male reproductive disorders may be symptoms of a common underlying fetal testicular dysgenesis, which may be caused by a disturbance in Sertoli cell and Leydig cell differentiation during fetal life leading to impaired testosterone production (Skakkebæk et al. 2001). Based on results from animal studies, shortening of the AGD in newborn males is attributed to impaired androgen action during a particularly sensitive developmental window, which is believed to correspond to gestational weeks 8 to 14 in humans (Welsh et al. 2008). The observation in this study of reduced AGD in boys, although statistically nonsignificant, is therefore consistent with an anti-androgenic effect of TCS on the fetus. This finding is also in accord with *in vitro* studies showing anti-androgenic action of TCS (Ahn et al. 2008; Gee et al. 2008), although a study examining developmental effects following *in utero* TCS exposure in rats did not show significant changes in AGD in male offspring (Axelstad et al. 2013), and no association was observed between prenatal triclosan exposure and undescended testis in a rather small study of 151 male newborns (Chevrier et al. 2012).

TCS is quickly metabolized with a urinary excretion half-life of < 24 hr (Sandborgh-Englund et al. 2006). A single spot urine sample collected in approximately gestational week 28 may therefore potentially fail to accurately reflect average fetal exposure during the entire pregnancy or during the developmental window early in fetal life that is suggested to be particularly sensitive for the formation of the AGD (Welsh et al. 2008). However, studies of temporal variability in urinary excretion of TCS have indicated reasonable temporal consistency in TCS excretion (Lassen et al. 2013; Meeker et al. 2013; Philippat et al. 2013), with interclass correlation coefficients among pregnant women between 0.47 and 0.58 (Meeker et al. 2013; Philippat et al. 2013) suggesting relatively stable exposure sources to TCS throughout gestation. Nevertheless, some exposure misclassification is to be expected when using a single urine sample collected during pregnancy for exposure classification. Such misclassification may lead to underestimation of an association between prenatal TCS exposure and AGD. The third trimester of pregnancy is characterized by rapid fetal weight gain, and urine samples collected in approximately the 28th week of gestation may therefore characterize a relevant window of exposure for fetal growth effects. In this study, AGD was measured at approximately 3 months of age. In a study by Thankamony et al. (2009), AGD measurements at 3 months of age have been shown to be weakly, though significantly, correlated with AGD measurements at birth in both boys (*r* = 0.30) and girls (*r* = 0.26).

In this study, a relatively large number of statistical analyses were performed, and some of our results may be chance findings resulting from multiple testing. We had a relatively limited sample size and, hence, limited statistical power for analyses of sex-specific associations between TCS exposure and growth measures. The suggested sex-specific differences in outcomes are therefore hypothetical and should be confirmed in other studies.

It is possible that selection bias affected the results. Only 43% of the eligible women participated, and participants were more often of Danish origin and were better educated than nonparticipants. The women included in this study had a lower prevalence of smoking during pregnancy (~4%) than nonparticipants (12%), who were women giving birth between 2010 and 2013 and living in the recruitment area of the Odense Child Cohort (Kyhl et al. 2015). The mean age at delivery in our study was, however, identical to the mean age of all Danish women giving birth in Denmark in 2011 (Danish Health and Medicines Authority 2012), and the median TCS concentration among the pregnant women in this study was comparable with levels observed in Danish children 6–11 years of age and in their mothers from samples collected in 2011 (median 0.46 ng/mL and 0.64 ng/mL, respectively) (Frederiksen et al. 2013b). The women in this study had no prior knowledge of their TCS exposure, which therefore is unlikely to have affected their willingness to participate. Although all estimates of associations were adjusted for relevant confounders, we cannot exclude the possibility of confounding by other factors associated with TCS and growth measures, such as co-exposure to other environmental chemicals or lifestyle factors. Therefore, our findings need to be confirmed in additional human studies.

## Conclusions

Prenatal TCS exposure was associated with reduced head circumference and abdominal circumference at birth and with a reduced AGD at 3 months of age in boys, although the last two findings did not reach statistical significance (*p* < 0.10). No associations between prenatal TCS exposure and birth outcomes and AGD were found in girls, although AGD was measured for fewer girls and the interactions between child sex and TCS were not statistically significant. Because of the suggestive sex differences, we speculate that our findings are compatible with an anti-androgenic effect of prenatal TCS exposure on fetal development in boys. However, our findings need to be confirmed in other populations, and the biological mechanisms should be elucidated.
